# Adrenaline auto injectors pharmacokinetic/pharmacodynamic studies and potential consequences for clinical practice

**DOI:** 10.1002/clt2.12323

**Published:** 2023-12-18

**Authors:** Margitta Worm, Adam T. Fox, Magnus Wickman, Johannes Ring, Motohiro Ebisawa, Guillaume Pouessel, Pete Smith

**Affiliations:** ^1^ Allergology and Immunology Department of Dermatology, Venereology and Allergology Charité – Universitätsmedizin Berlin Germany; ^2^ Children's Allergy Service Guy's & St Thomas' Hospitals NHS Foundation Trust London UK; ^3^ Department of Environmental medicine Karolinska Institutet Hedetorp Sweden; ^4^ Technical University Munich (TUM) Department Dermatology Allergology Biederstein Berlin Germany; ^5^ National Hospital Organization Sagamihara National Hospital Sagamihara Japan; ^6^ Department of Pediatrics Allergology Unit Children's Hospital CH Roubaix and Pneumology and Allergology Unit Children's hospital Lille France; ^7^ Department of Clinical Medicine Griffith University School of Medicine Southport Queensland Australia

**Keywords:** adrenaline autoinjectors, anaphylaxis, pharmacodynamics, pharmacokinetics, skin‐to‐muscle distance

## Abstract

**Background:**

Anaphylaxis is a sudden multisystem allergic reaction which may result in a fatal outcome if not treated promptly. Guidelines worldwide suggest intramuscular adrenaline as the first‐line treatment for anaphylaxis outside a perioperative reaction. Adrenaline autoinjectors (AAIs) are widely used self‐administrable devices, especially in community settings. Different commercial AAIs have been authorized to be marketed in Europe. For an AAI to be efficacious, a rapid adrenaline delivery in patients, including those who are overweight or obese, resulting in an optimal cardiovascular (CV) response, is a key feature. AAIs are designed to achieve this requirement, which is reflected in their differing functional properties such as primary container selection, drug delivery mechanism (cartridge‐or syringe‐based), needle length, needle gauge, and adrenaline dose (150 μg, 300 μg, or 500 μg). However, the differences in functional properties across these devices may play a critical role in achieving these requirements as well as the differences in ergonomics in the handling of these devices.

**The purpose of this review:**

Considering the dynamic pharmacokinetic/pharmacodynamic (PK/PD) profiles of different AAIs marketed in Europe and their effect on adrenaline delivery, the expert panel, also serving as author for this paper have carried out a detailed analysis of the PK/PD profiles of four AAIs, namely, Anapen, Emerade, EpiPen, and Jext, to delineate the adrenaline delivery and their subsequent physiological effects on the backdrop of device characteristics, dose strength, and the skin‐to‐muscle distances of the participants.

## 1 INTRODUCTION

Anaphylaxis is a potentially life‐threatening systemic hypersensitivity reaction. Severe anaphylaxis is mainly characterized by breathlessness and/or compromise in circulation and may occur without typical skin features.^1^, ^2^ Anaphylaxis presents as an emergency and even a few minutes of delay in treating anaphylaxis can lead to death.^3^, ^4^ Food (e.g., peanut, tree nuts, milks, shellfish), insect venom (e.g., wasps, honeybees), and drugs (e.g., antibiotics, analgesics) are among the most common triggers of anaphylaxis.^5^, ^6^, ^7^ It is most often unpredictable and may occur in community settings, such as restaurants, schools, or during leisure.^8^ Hence, people at risk of severe allergic reaction and their caregivers should have an easily accessible emergency kit at all times. A treatment that can be rapidly self‐administered is generally agreed to reduce morbidity and mortality, as well reduce anxiety regarding accidental exposure.

Intramuscular (IM) injection of adrenaline in the mid‐anterolateral (AL) thigh is the first line of treatment for anaphylaxis recommended using the international guidelines (World Anaphylaxis Organization, WAO 2020) outside the perioperative setting where intravenous adrenaline is recommended.^1^ The premeasured adrenaline autoinjectors (AAI) are easy to use, self‐injectable emergency response devices that ensure prompt adrenaline delivery and hence are considered as life‐saving emergency treatment for anaphylaxis.^9^ They reduce the possibility of dosing errors (overdosing/underdosing) that may arise due to manually drawing medications via a syringe or assembling ampoule/needle/syringe, especially in a panicked emergency situation.^9^, ^10^ However, the stability and sterility of adrenaline prefilled syringe depends on the storage condition and is questionable beyond 3 months after manufacture.^11^

Four AAIs, namely Anapen (Bioprojet), Emerade (Bausch and Lomb), EpiPen (Mylan Specialty L.P), and Jext (ALK‐Abello Ltd) are currently authorized to be marketed in Europe. Going further, the AAIs presented in the article, that is, Anapen, Emerade, EpiPen, and Jext will be referred to as Product A, EM, EP, and J, respectively. The functional features of these AAIs are summarized in Table 1.

**TABLE 1:** Functional features of the analyzed AAIs.

Properties	Product A^12^	Product EM^13^	Product EP^14^	Product J^15^
Needle length	10 mm ± 1.5 mm	For 150 μg: 16 mm For 300 μg: 23 mm For 500 μg: 23 mm	∼15 mm	150 μg–13 mm 300 μg–15 mm
Drug delivery mechanism	PFS (syringe based)	PFP (syringe based)	PFP (cartridge based)	PFP (cartridge based)
Indications	Indicated in the emergency treatment of severe allergic reactions (anaphylaxis) to foods, insect stings or bites, drugs, and other allergens, as well as idiopathic or exercise induced anaphylaxis^12^, ^13^, ^14^, ^15^			

Abbreviations: mm, millimeter; PFP, prefilled pen; PFS, prefilled syringe; μg, microgram.

The currently available AAIs cannot be deemed as ‘ideal’ since there is variability in their functional features.^9^ The clinical importance of any AAI is characterized by its ability to be robust and reliable enough to withstand real‐life use and rapidly deliver relevant dose strengths to the correct tissue compartment within the right timeframe. It must also be easy, convenient, and safe for patients or caregivers to use.^3^, ^16^ The factors that govern the clinical significance and adrenaline delivery through an AAI are summarized in Table 2.

**TABLE 2:** Factors governing clinical significance and adrenaline delivery through an AAI.

Factors governing clinical significance of an AAI	Factors governing adrenaline delivery through an AAI
•Ability to deliver relevant dose strengths of adrenaline	•Propulsive force determining adrenaline velocity through the needle and beyond the needle tip upon tissue penetration.
•Ability to deliver adrenaline in correct tissue compartment	•Injection angle
•Ability to deliver adrenaline within the right timeframe	•SC fat thickness
•Easy, convenient, and safe to be used by patients and caregivers	•Device delivery mechanism (cartridge ― or syringe ―based)

Abbreviations: AAI, adrenaline autoinjector; SC, subcutaneous.

One of the common concerns is sub‐cutaneous (SC) adrenaline delivery in obese individuals due to longer skin‐to‐muscle distance (STMD). Sub‐cutaneous injections can potentially result in lower peak plasma concentration (C_max_) compared with IM syringe, leading to a sub‐optimal adrenaline delivery into the target tissue compartment.^17^, ^18^ Another concern is the risk of the AAI needle injection into the bone in children under 15 kg weight, especially with devices with longer needle length.^19^

Previous studies involving Product EP and IM syringe allowed a rapid systemic adrenaline delivery versus SC injection in adults and children with a history of anaphylaxis. However, in both the studies, participants' STMDs were not factored during pharmacokinetic (PK) assessments.^20^, ^21^ Subsequent *ex‐vivo* penetration studies (with fresh pork shoulder) demonstrated a deeper penetration with Product EP and Product J compared with Product A.^16^, ^22^ Until recently, STMDs of the participants were considered, only to determine suitable needle lengths for AAIs to achieve IM adrenaline delivery with avoidance of intraosseous/periosteal injection.^23^ Considering that STMD impacts adrenaline delivery, a thorough PK and pharmacodynamic (PD) profiling in individuals with different STMDs can provide an understanding of adrenaline delivery, distribution, exposure, and physiological activity. Furthermore, PK/PD results can help in understanding the effect of inter‐device functional differences and participants' STMDs on the adrenaline delivery.^24^ Hence, the Committee for Medicinal Products for Human Use (CHMP) recommended PK/PD studies for each AAI that are marketed in Europe to be performed by their respective Marketing Authorization Holder (MAH).^25^

In this paper, the findings of the three PK/PD studies for Products A,^26^ EM,^27^ and EP,^28^ and the PK data for Product J^29^ published in a review article through a freedom of information request from Medicines and Healthcare products Regulatory Agency (MHRA) in the United Kingdom are analyzed. Our data will support clinicians in making evidence–based decisions, and optimal treatment effectiveness by maneuvering the inter–device functional differences in each AAI.

## 2 METHODS

Data from three PK/PD studies on Products A,^26^ EM,^27^ and EP^28^ and one review article with PK data on Product J^29^ were analyzed in a comparative manner. Key information and data with respect to device characteristics (including needle length and drug delivery system), PK/PD parameters, and final findings have been extracted. The methodology used to assess PK/PD of all the 4 AAIs is summarized in the following sections. Owing to very little information pertaining to Product J available in the public domain, a detailed methodology could not be included in this paper.

### 2.1 Product A

An open label, randomized, crossover study by Duvauchelle et al. compared the PK/PD of adrenaline administered via Product A versus prefilled IM syringe.^26^ The study participants included adult men weighing normal and overweight women with low and moderate STMDs, respectively (Supplementary Table 1). Low STMD participants were crossed over to receive one injection of adrenaline, each via Product A (300 μg adrenaline) and prefilled IM syringe (300 μg adrenaline). The same group of participants also received a higher adrenaline dose only via a prefilled IM syringe (500 μg adrenaline). Similarly, moderate STMD participants were injected with adrenaline via Product A (300 μg adrenaline). In low STMD participants, Product A was injected at 2 sites, that is, mid‐ and inferior‐AL parts of the thigh muscle, while prefilled syringe was injected only in the mid‐AL part. In moderate STMD participants, Product A was injected at only one site, that is, inferior‐AL part of thigh muscle. Prefilled IM syringes had a longer needle versus Product A (25.4 vs. 10.5 mm, respectively). Ultrasound was used to capture ultrasonic images of participants' STMDs as well as injectate depot depths. Blood was sampled at pre‐determined time intervals during both pre‐and post‐dosing. Systolic blood pressure (SBP) and heart rate (HR) measurements were performed sequentially before and after each injection.^26^

### 2.2 Product EM

An open‐label, four‐way crossover study assessed the PK/PD of Product EM in 2 different doses versus Product EP and Product J (NCT03282929).^27^, ^30^ The study was divided into 2 parts, Part 1 and Part 2; Part 1 of the study involved a comparison between SC and IM injections without any relevant comparison between the AAIs. Since the sample size was less (*n* = 8), no legible conclusions can be drawn from the results. Hence, discussions pertaining to Part 1 are excluded from this paper.

The study included participants with low, moderate, and high STMDs (Supplementary Table 1). While the low and moderate STMD groups included both men and women, the high STMD group comprised only women. Each participant received four adrenaline injections via three AAIs as per the respective device instructions and two doses were administered. The adrenaline administered via Product EM was dosed at 300 and 500 μg, while adrenaline administered via Product EP and Product J was dosed at 300 μg each, respectively. The needles used for Product EM were relatively longer versus Product EP and Product J (23 vs. 16 mm and 15 mm, respectively). Each injection was separated by a 24‐h washout period. Blood analysis, BP, pulse rate, and electrocardiogram (ECG) assessments were performed during both occasions, that is, pre‐and post‐dosing at regular intervals.^27^, ^30^

### 2.3 Product EP

An open‐label, randomized, three–way crossover studies by Worm et al. compared the PK/PD of adrenaline administered via Product EP versus IM syringe.^28^ Adult men and women with low, moderate, and high STMDs were enrolled. Each participant was crossed over to receive 3 adrenaline injections on the mid–AL part of the thigh muscle via Product EP and IM syringe (each containing 300 μg) or saline via IM syringe (Supplementary Table 1). It should be noted that the needle length for IM syringe was 30% longer than that of participants' mean STMD (at minimum compression to ensure that adrenaline was indeed delivered IM), intending a fair comparison between the two devices. Additionally, all participants with a skin–to–bone distance (STBD) ≥20 mm received a fourth adrenaline injection (300 μg) in the distal–AL part of the thigh muscle. Participants with STBD <20 mm and low STMD did not receive the fourth injection. All the participants underwent a 24‐h washout period between each of the injections. To determine the PK parameters, the blood sample was withdrawn and analyzed at regular time intervals. The PD parameters were blood pressure (BP, systolic and diastolic blood pressure [SBP and DBP] respectively) and heart rate (HR) measurements. All the assessments were done at regular time intervals, pre– and post–injection.^28^

### 2.4 Product J

In a review article, Turner et al.^29^ presented the PK data of adrenaline administered via a Product J or IM syringe as part of the manufacturer's study. As mentioned in the methodology of this review article by Turner et al., limited data on Product J was available in the public domain; therefore, PK data on Product J was obtained from MHRA through a freedom of information request. The study participants in the manufacturer's study on Product J included healthy adult participants belonging to low, moderate, and high STMD categories, who were crossed over and received adrenaline via Product J and IM syringe (dose for both devices; 300 μg). While the needle length for Product J was reported to 15 mm, the needle lengths for IM syringe could not be recorded from the available literature except that longer needle was used for IM syringe in participants with high STMD to ensure IM delivery of adrenaline. Blood was sampled during both pre‐dosing and post‐dosing for 180 min^15^, ^29^

## 3 RESULTS

### 3.1 Product A

**FIGURE 1:**
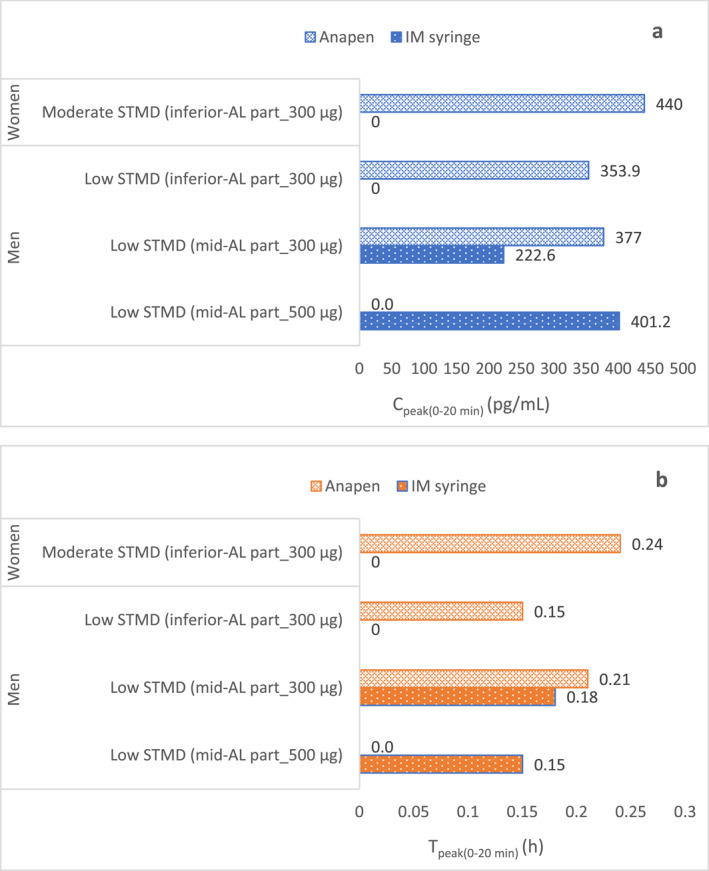
Comparison between PK parameters measured in terms of C_peak (0‐20 min)_ and T_peak (0‐20 min)_ of adrenaline injected via Product A (Anapen) and intramuscular (IM) syringe in mid or inferior anterolateral (AL) part of the thigh in men with low skin to muscle distance (STMD) and women with moderate STMD^26^ (A). C_peak (0‐20 min)_ of adrenaline via Product A (Anapen) and IM syringe (B). T_peak (0‐20 min)_ of adrenaline via Product A (Anapen) and IM syringe. T_peak (0‐20 min)_ (h) is expressed as mean value.

In the study by Duvauchelle et al.,^26^ the peak adrenaline concentrations achieved via Product A were measured for only the first 20 min post‐injection (C_peak0‐20 min_). Product A led to a higher C_max_ versus IM syringe within the first 20 min post–dose in low STMD participants (377.0 pg/mL vs. 222.6 pg/mL). In moderate STMD participants, Product A displayed a C_max_ of 440.0 pg/mL. In low STMD participants, the corresponding AUC_0‐20 min_ was greater with Product A versus IM syringe (300 μg). The T_peak 0‐20 min_ values measured over the first 20 min post‐injection did not differ between Product A and prefilled IM syringe (300 and 500 μg) in low STMD participants. However, when Product A was injected in moderate STMD participants, there was a slight delay in achieving T_max_ over the first 20 min post–injection compared with low STMD participants (Figure 1).^26^

A higher C_max_ was observed for 500 μg prefilled IM syringe versus Product A in a lower dose that is, 300 μg when injected in low STMD participants. In moderate STMD participants, adrenaline was not administered via a prefilled IM syringe (neither 300 μg nor 500 μg) at all. Hence, PK/PD parameters could not be compared between Product A and IM syringe in this group. The ultrasonic imagery of injectate droplets from Product A measured the depth of the fluid depot, which was found to be ˗6–18 mm. This depth was greater than the maximum thickness of skin in low STMD participants measured at the mid‐AL thigh, that is, 10.3 and 6.3 mm at the inferior AL of the thigh muscle, respectively. The average skin thickness in moderate STMD participants at the mid‐AL thigh was 15.8 mm. Interestingly, in these participants, the average depth of the depot formed by injectate droplets was 11.5 mm. This depth was quite close to the needle length of Product A (10.5 mm). Another open‐label study conducted in 54 healthy participants showed that Product A 500 μg injection produced a rapid increase in circulating adrenaline levels, irrespective of the body mass index (BMI; normal, overweight, and obese) and site of injection (IM or SC). A significant increase in SBP and HR was also observed.^31^

Elevations in HR were seen with both Product A and IM syringes, with an approximate increase of 17 beats/minute. However, HR elevation was rapid and higher with Product A versus prefilled IM syringe when measured within the first 20 min post‐dosing in all participants. The magnitude of HR elevations with Product A was similar at both injection sites. Therefore, when measured within the first 20 min post–injection, Product A achieved C_max_ and AUC that was nearly two times higher versus the prefilled IM syringe.^26^ The PK parameters of Product A and IM syringe measured in the tested population are detailed in Supplementary Table 2.

### 3.2 Product EM

**FIGURE 2:**
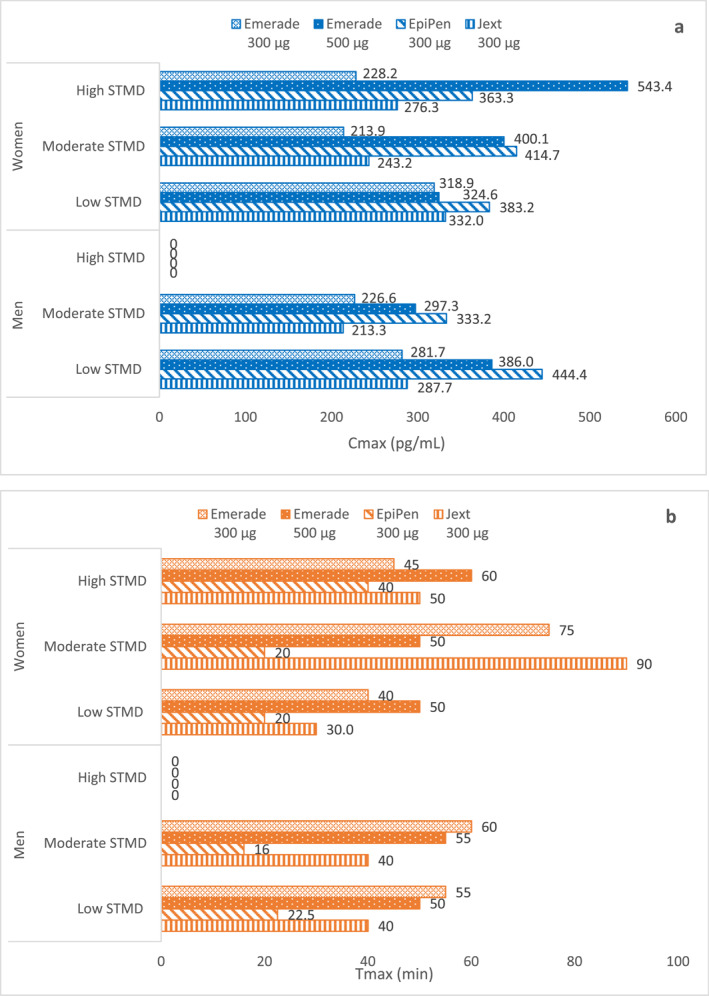
Comparison between PK parameters measured in terms of C_max_ and T_max_ of adrenaline injected via Product EM (Emerade) 300 μg and 500 μg, Product EP (EpiPen) 300 μg, and Product J (Jext) 300 μg in mean and women with low, moderate, and high STMDs^27^ (A). C_max_ of adrenaline via Product EM (Emerade) 300 μg and 500 μg, Product EP (EpiPen) 300 μg, and Product J (Jext) 300 μg (B). T_max_ of adrenaline via Product EM (Emerade) 300 μg and 500 μg, Product EP (EpiPen) 300 μg, and Product J (Jext) 300 μg.

As part of a single dose, open‐label, crossover study, the PK/PD of adrenaline administered by Product EM 300 μg, Product EM 500 μg, Product EP, and Product J was assessed in healthy men and women.^27^, ^30^ The plasma adrenaline concentration administered with all the 4 AAIs (Product EM 300 μg, Product EM 500 μg, Product EP, and Product J) was measured within 5 min post‐dosing.^27^ All AAIs demonstrated a rapid increase in adrenaline plasma concentration within 5 min post–injection. However, the highest increase in plasma levels was observed with Product EP (210.7 pg/mL) followed by Product D (150.2 pg/mL). Product EM when administered in 300 and 500 μg doses resulted in a plasma adrenaline concentration of 98.2 pg/mL and 145.4 pg/mL, respectively. In male participants with low and moderate STMD and female participants with moderate STMD, C_max_ was highest with Product EP followed by Product EM 500 μg, followed by Product J and Product EM 300 μg (Figure 2). In female participants with high STMD, Product EM 500 μg displayed the highest C_max_ values, followed by Product EP, Product J, and lastly Product EM 300 μg. Since the high STMD group included only female participants, a comparison with male participants with high STMD could not be made. Moreover, higher dosing with Product EM 500 led to higher C_max_ but did not increase the availability of adrenaline in the critical first 10 min. Furthermore, Product EP presented the highest AUC measured until 10 min (AUC_0‐10 min_) across all the participants versus Product EM 300 and 500 μg and Product J. A delayed T_max_ was observed with both Product EM 300 and 500 μg followed by Product J across all participants. Product EP, on the contrary, showed a shorter T_max_ across all participants versus Product EM 300 and 500 μg and Product J. An intra‐device dosing difference in C_max_ was observed for both the doses of Product EM (C_max_ range [300 μg], 220.2 pg/mL–298.5 pg/mL vs. [500 μg], 341.5 pg/mL–543.4 pg/mL, respectively). Although HR and BP were measured, results did not correlate with the adrenaline exposure and remained inconclusive. Overall, Product EP displayed a rapid and higher systemic adrenaline delivery in the initial crucial period, followed by Product EM 500 μg, Product J, and Product EM 300 μg. Supplementary Table 3 presents a summary of the PK parameters of adrenaline administered via Product EP, Product EM 300 and 500 μg, and Product J.

### 3.3 Product EP

**FIGURE 3:**
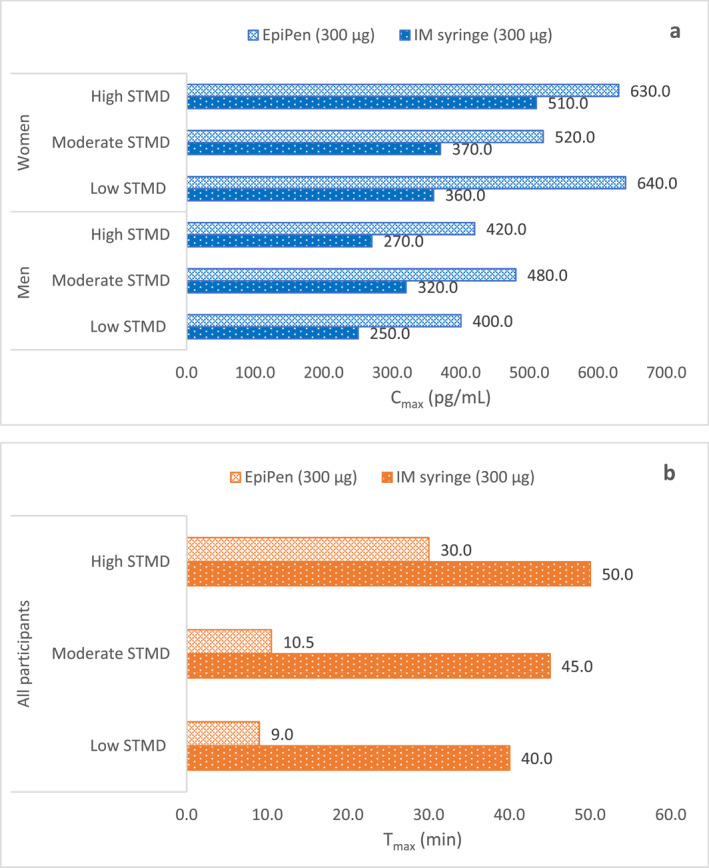
Comparison between PK parameters measured in terms of C_max_ and T_max_ of 300 μg adrenaline injected via Product EP (EpiPen) and intramuscular (IM) syringe in men and women with low, moderate, and high skin to muscle distance (STMD) in mid‐anterolateral (AL) thigh. (A) C_max_ of adrenaline via Product EP (EpiPen) and IM syringe.^28^ (B) T_max_ of adrenaline via Product EP (EpiPen) and IM syringe. T_max_ for Product EP (EpiPen): Presented as median values of all participants without gender stratification.

As part of an open‐label, randomized, crossover study, the PK/PD of adrenaline administration via Product EP or IM syringe in participants with varying STMD has been assessed previously.^28^ A rapid increase in adrenaline plasma concentration was observed with Product EP across participants with a wide range of STMD marked by a shorter T_max_. Overall, women participants across all STMD categories achieved a higher C_max_ versus men when administered adrenaline via Product EP (C_max_ range 520–640 pg/mL vs. 400–480 pg/mL, respectively) (Figure 3). The area under the curve (AUC) for adrenaline calculated from time zero to first 30 min (also called as partial AUC, pAUC) was greater with Product EP versus IM syringe. This trend was observed across all STMD groups (geometric mean ratio [GMR]: low STMD, 2.09; moderate STMD, 1.64; high STMD, 2.90). Within the Product EP group, T_max_ was delayed for participants with higher STMD versus those with moderate and low STMD, that is, 30 min versus 10.5 min and 9 min, respectively). However, adrenaline delivered via IM syringe (with needle length 30% longer than participants' mean STMD at minimum compression) required a longer time to achieve C_max_ (T_max,_ 50 min). Across all participants, Product EP, administered at the distal AL thigh had a slightly delayed T_max_ versus mid AL thigh (T_max;_ 25 vs. 20 min). When compared with IM syringe, Product C administered at distal‐AL thigh still displayed a rapid T_max_ (50 vs. 25 min). The PK parameters of adrenaline administered via the product EP and IM syringes are summarized in Supplementary Table 4.

In all participants, adrenaline administration via both the Product EP and IM syringes displayed HR elevations within 5 min post–injection. These elevations were observed when adrenaline reached a plasma threshold level of 100 pg/mL with both the Product EP and IM syringes. Product EP displayed greater and rapid HR elevations versus IM syringe at the mid‐AL thigh (maximum HR, 76.9 beats/min vs. 72.6 beats/min [geometric mean ratio, 1.05; 95% CI 102.2%–108.4%] and shorter time to maximum HR, 33.8 vs. 60.4 min [geometric mean ratio, 0.66; 95% CI 20.8%–88.6%]). Compared to the IM syringe, Product EP rapidly delivered a higher adrenaline systemic concentration in all participants, including those with high STMD.

### 3.4 Product J

**FIGURE 4:**
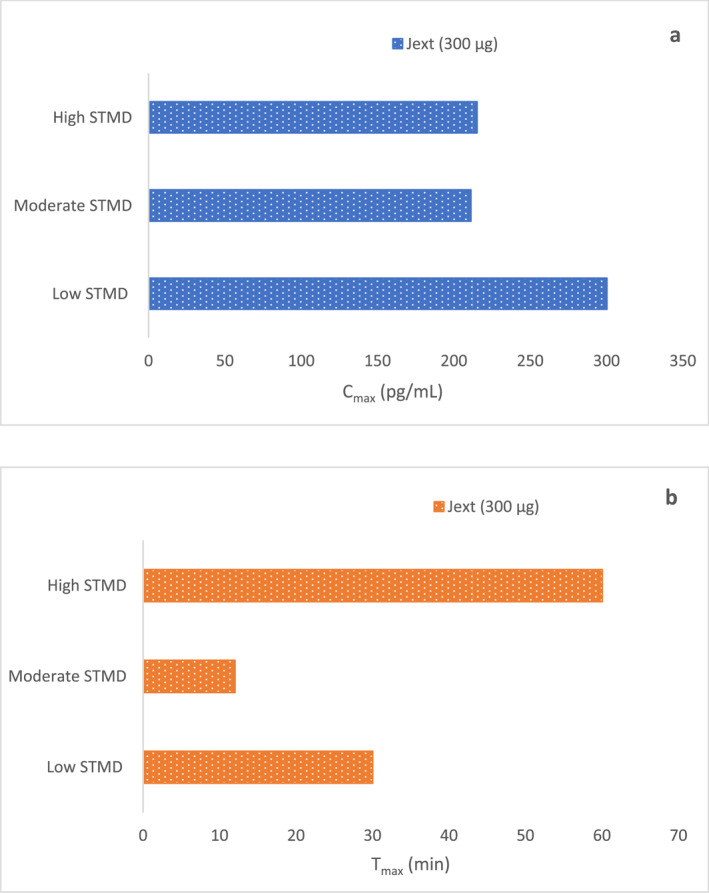
(A) C_max,_ and (B) and T_max_ of 300 μg adrenaline injected via Product J (Jext) in the low, moderate, and high skin to muscle distance (STMD) participants.^29^

As per the data from the manufacturer's study on Product J presented in a review article by Turner et al.,^29^ the value of C_max_ displayed by low STMD participants (<15 mm) was higher versus moderate (15–20 mm) and high STMD (>20 mm) participants (300.0 pg/mL vs. 211.0 pg/mL and 215.0 pg/mL, respectively) (Figure 4). Likewise, the median T_max_ in high STMD participants was higher than that in moderate and low STMD participants (60 vs. 12 min and 30 min, respectively). The decreasing AUC_0‐30 min_ values across moderate and high STMD participants revealed 27% and 64% lower adrenaline absorption, reflecting a delay in overall absorption kinetics with increasing STMD. Compared to IM injection, mean plasma adrenaline concentration measured in high STMD participants were the following in 8 min (AUC_0‐8 min,_ 0.39 [90% Confidence Interval, CI 0.20–0.75]), 16 min (AUC_0‐16 min,_ 0.56 [90% CI 0.31–0.99]) and 30 min (AUC_0‐30 min_, 0.66 [90% CI 0.39–1.12]).^29^ A short summary of the PK parameters administered through Product J and IM syringe is presented in Supplementary Table 5.

## 4 DISCUSSION

Over the last 4 decades, autoinjector technology has been widely used to deliver life–saving adrenaline during an episode of anaphylaxis.^24^ The PK/PD profile of adrenaline depends on the AAI used for its delivery.^32^ This paper is an attempt to better understand the impact of the devices' functional properties on systemic adrenaline delivery and its subsequent CV response due to the PK/PD profile. Throughout the studies, a syringe‐based delivery was a common comparator to the AAIs as adrenaline administered via a syringe and needle is the standard clinical practice. Product EP and Product A were directly compared with IM syringe that acted as internal standard (similar to non‐inferiority trials) in these studies.^26^, ^28^ Additionally, a placebo was included as one of the comparators against Product EP.^28^ Product EM has directly been compared with Product EP and Product J.^27^ Since the adrenaline concentration necessary to counteract the anaphylactic symptoms is unknown, a risk‐benefit assessment needs to be ascertained. The data so far are limited and constrained in some ways, and hence need to be assessed carefully before deployment in clinical practice. The study results of Product A, Product EM (NCT03282929), Product EP, and Product J have been discussed individually in the sections below.

### 4.1 | Product A

Product A achieved a higher systemic concentration versus prefilled IM syringe in low STMD participants during the first 20 min post–dose (Supplementary Table 2). Though similar results were achieved in moderate STMD participants with Product A, it was not compared with the IM syringe. Product A achieved higher elevations in HR versus IM syringe within 10 min post‐dose in low STMD participants, which is of more clinical relevance owing to the fact that anaphylactic treatment is highly time sensitive.^26^ The HR changes clearly corresponded to changes in plasma adrenaline levels, which is reflected in Product A achieving favorable CV response. Product A further demonstrated a favorable PK/PD profile in spite of having a short needle length versus IM syringe. Additionally, the adrenaline depot depth formed by Product A was close to its needle length, suggesting that moderate STMD participants received the drug in the SC compartment instead of IM. Despite this, Product A achieved a higher systemic adrenaline concentration versus a prefilled IM syringe that had the needle long enough to reach the muscles.

Product A showed consistent adrenaline delivery in low and moderate STMD patients; however, a direct comparative study with IM syringe in moderate STMD participants was not carried out. Moreover, the PK parameters of Product A in high STMD (>20 mm) remain unknown as this group was not included in the study. Hence, there are certain knowledge gaps that exist with respect to the efficacy of Product A in obese populations. Moreover, direct comparisons of Product A and IM syringe penetration studies revealed that fluid depot depth injected via Product A did not exceed the needle length, and the injectate remained in the SC layer in moderate STMD participants. It will be interesting to study whether Product A can achieve a consistent adrenaline delivery when moderate STMD participants are replaced by high STMD participants. Schwirtz *et al*. compared the penetration depth of Products A, EP, and J using pork shoulders. They found that adrenaline delivered via Products EP and J reached the muscles even when STMD was greater versus their respective needle lengths. In contrast, adrenaline injected via Product A could not be delivered deeper than its needle length.^16^

### 4.2 | Product EM

The data comparing PK data of both doses of Product EM (300 and 500 μg) with Product EP and Product J showed that Product EP consistently achieved rapid systemic delivery with higher AUC_0‐10 min_ compared to both the doses of adrenaline injected via Product EM. This can be of great clinical relevance given the short time period for treating an anaphylaxis episode.^27^ Furthermore, Product EM (300 μg) showed slower adrenaline absorption across all the participants, including those with high STMD. Although longer needles were used in Product EM versus needles used in Product EP and Product J, the former could not achieve rapid and higher adrenaline concentrations in all participants that emphasizes the pivotal role of the device in optimal adrenaline delivery. Product EM uses a syringe–based mechanism for delivering the adrenaline systemically. The force of the injection spring may be hindered by the glass syringe, thereby delivering sub–optimal amount of adrenaline (Table 1).^3^ With respect to intra‐device dosing C_max_ difference, it is apparent that Product EM might require a higher adrenaline dose to achieve higher systemic concentration. However, both doses of adrenaline delivered via Product EM achieved a lower systemic adrenaline concentration versus Product EP in the initial crucial period, which could be attributed to the different drug delivery mechanisms (Supplementary Table 3).

Although some patients may require higher (500 μg dose) and repeated adrenaline doses, the studies‐citing the advantage of higher doses are limited. As per the expert statement of the World Allergy Organization (2008), a second dose is reported to be required in 16%–36% of patients.^33^ However, high doses may increase the risk of dose accumulation after 40–50 min if a second 500 μg dose was to be injected in rapid succession. Overall, more studies are required to understand the impact of two immediate 500 μg dose in normal and obese individuals in real–life settings.

In summary, the PK results of Product EM were highly variable and hence, the PD outcome remained inconclusive. The adrenaline absorption from Product EM was also found to be slower with inadequate response in participants with high STMD.^27^, ^31^

### 4.3 Product EP

Product EP led to a rapid and higher adrenaline concentration versus IM syringe across all STMD participants (low, moderate, and high) (Supplementary Table 4). Higher systemic concentrations with Product EP remained independent of the injection sites in both the mid‐ and distal AL thigh even when the needle length was shorter than that used with the high STMD participants. This is clinically relevant, especially in the obese population that forms an important patient subset.^28^ In a separate study by Song et al. conducted in cadaver pig legs, an adrenaline depth of approx. 27.8 mm was achieved with Product EP with a needle length of 14.3 mm (94.4% beyond the needle length).^34^, ^35^ This could be achieved by two factors namely, needle penetration depth and the adrenaline propulsion force.^3^ Deeper needle penetration could be achieved with a combination of extended needle length and activation force (for Product EP, 17.0 mm and 8.0 pounds, respectively) that compresses the tissues, decreases the STMD by 25% in women and 19% in men, respectively.^3^, ^36^ Additionally, based on the study conducted in ballistic gelatine, the results reveal that higher activation force with Product EP warrants higher adrenaline volume into the muscles (0.22 mL out of total 0.3 mL dose, 74.3%), compared to a lower activation force as seen in Product A (∼0.08 mL out of total 0.3 mL dose, 25.7%).^3^, ^19^ Although the effects have not been tested in clinical settings, the high activation force should be considered while ensuring sufficient adrenaline delivery.^3^ In another study conducted in marbleized beef (containing varying amounts of IM fats) > 95.9% of adrenaline was absorbed into the muscle tissue within 1 s post‐injection.^37^ The greater adrenaline propulsion with Product EP can be explained by its lower needle gauge (22 Ga; with large needle diameter), syringe diameter, stopper friction, and force driving the stopper.^38^ The greater speed and higher dispersion of adrenaline in the vascular bed leads to rapid absorption and systemic uptake.^3^, ^28^ Interestingly, there are reports that *fascia lata* surrounding the deep tissues in the thigh might act as an impervious barrier to adrenaline delivery to the muscles.^39^, ^40^ Although, a study using cadaver pig legs concluded the inability of Product EP in breaching the *fascia lata* barrier and reaching the muscular layer, the fact that even an SC injection reaches systemic circulation points either toward chance penetration in one of the intermittent blood vessels supplying the facial plane or greater tissue compression and propulsion force.^19^, ^38^, ^39^, ^40^

This paper highlights that in healthy individuals adrenaline is delivered to reach desired plasma levels; however, there can be a wide variability in T_max_ and plasma levels depending on the needle length and the amount of subcutaneous fat.^28^ Given the nature of anaphylaxis, efforts must be made to ensure that at least the recommended adrenaline dose reaches the target tissue.^41^ This becomes even more essential when the adrenaline concentration necessary to counteract anaphylactic symptoms is unknown.^42^ Hence, all AAIs aim to rapidly deliver an optimal adrenaline dose into the thigh muscle for systemic availability.

### 4.4 Product J

It is interesting to note that plasma adrenaline exposure achieved by Product J was inferior to IM syringe up to 30 min post‐dosing in high STMD participants.^29^ This was in stark contrast with Product EP, which even with delayed absorption achieved higher plasma adrenaline concentration similar to internal standard, that is, IM syringe used in high STMD participants. Although the functional parameters of both the devices, Product J and Product EP, including the extended needle length and the delivery system (cartridge‐based) bear similarities, the PK profile of Product J is similar to that of syringe‐based Product EM.^27^, ^28^ Furthermore, these results were also consistent with PK data obtained on comparing both the doses of Product EM (300 and 500 μg) with Product EP and Product J, wherein Product J displayed delayed adrenaline absorption compared to Product EP and Product EM 500 μg. With limited PK data, analyzing potential factors leading to these conflicting results is relatively challenging and represents an important knowledge gap that needs immediate attention. While some confounding factors related to the devices themselves and their handling can affect the ultimate adrenaline delivery from the device, researching these factors is of utmost importance.

## 5 CONCLUSIONS: SUMMARY AND CLINICAL IMPLICATIONS

In summary, this review analyzes the PK/PD results of the 4 AAIs in healthy individuals with varying STMD. Although the results were obtained from observations made in healthy individuals, this review serves as an opportunity to showcase the best achievable PK/PD in patients experiencing anaphylaxis that are likely to have compromised drug absorption and circulation. The current data also highlight factors that determine rapid and optimum systemic adrenaline delivery. The responses of the authors to practical questions pertaining to key determining factors for adrenaline delivery and their corresponding clinical significance are presented in Table 3. While none of the existing AAIs are ideal in all aspects, it is important to recognize that factors such as drug delivery mechanisms, dose strength, and individual STMDs play a crucial role. The current data suggest that needle length is not an absolute parameter to ensure adequate adrenaline delivery and AAIs with shorter needles are able to deliver the optimal dose of adrenaline systemically. Although BMI was thought to be an important surrogate, this hypothesis is not supported by the data presented. Furthermore, even though no accurate dosing has been established to manage anaphylaxis, 300 μg adrenaline is proven to be safe over many years. Nonetheless, more studies on this topic and high‐quality evidence are essential to understand the right dosing scheme to manage anaphylaxis.

**TABLE 3:** Expert opinion on key determining factors governing adrenaline delivery and their corresponding clinical significance.

Key determining factors governing adrenaline delivery	Clinical significance
• Needle length:	Needle lengths shorter than the STMD of an individual can provide optimal bioavailability and corresponding CV responses with the aid of delivery force compensation by spring loaded mechanism Needle lengths greater than the STMD of an individual can hit the bone and cause laceration especially in pediatric population owing to needle hooking at the tip or breaking‐off
Not the only critical parameter to ensure rapid and adequate adrenaline delivery in obese population	
• Need to look beyond delivery mechanism:	The clinicians may refrain from resorting to ‘one size fits all’ approach while selecting the appropriate autoinjectors and use a ‘device by device’ approach. They may evaluate the BMI and obesity parameters of the individual while prescribing the right device.
Apart from the activation force, and drug delivery mechanism used by the device, adrenaline delivery and penetration depth are governed by needle gauge, activation force, propulsive force of adrenaline through the needle, syringe diameter, stopper friction, driving force, volume of adrenaline released upon device activation and subcutaneous (SC) fat thickness.	
• Dosing dependence is related to device mechanism:	Clinicians may resort to using lower dose of adrenaline, that is, 300 μg using cartridge–based devices and overcome the risk of overdosing with higher doses, that is, 500 μg, as adrenaline has a small therapeutic window. The higher doses may be reserved for morbidly obese individuals (>100 kg)
Although using a lower dose, cartridge–based devices can achieve clinically relevant serum concentration versus syringe–based devices that use similar or even higher dose.	
• No critical advantage of higher dosing:	Although higher doses of adrenaline, that is, 500 μg cannot be ruled out, only limited individuals require a second higher dose during initial minutes of anaphylaxis. Clinicians may adopt ‘device‐by‐device’ approach while using two high doses in quick succession to prevent adverse cardiac events such as Takotsubo cardiomyopathy
There is no critical advantage with higher dose using auto injector devices in the initial minutes of anaphylaxis.	

Abbreviations: BMI, body mass index; CV, cardiovascular; STMD, skin to muscle distance.

## 6 LIMITATIONS

There are certain limitations to the papers that were used as the major base for this review. Firstly, the PK/PD data were assessed in healthy participants and not in participants undergoing anaphylaxis reaction. However, ethically, it is challenging to conduct the study in indicated population as prompt adrenaline administration is critical for an individual's survival.^43^ Secondly, not all AAIs have been tested in all STMD groups or genders nor are all of the data made publicly available. Thirdly, HR and BP measurements have been the only parameters used to measure the PD aspect of the AAIs. Measuring mean arterial pressure (MAP) and stroke volume (SV) may serve as additional PD parameters that may further support AAIs' efficacy.^44^ Fourthly, there is a lack of studies reporting PK data on second doses of AAI and the risk of cardiotoxicity, particularly with the devices used for administering 500 μg dose, especially Product A wherein a late serum peak occurs. Finally, comparator–controlled trials with a large sample size are warranted in the near future to confirm these findings.

## AUTHOR CONTRIBUTIONS

**Margitta Worm**: Conceptualization; (Equal), Writing – original draft; (Equal), Writing – review & editing; (Equal). **Adam T. Fox**: Conceptualization; (Equal), Writing – original draft; (Equal), Writing – review & editing; (Equal). **Magnus Wickman**: Conceptualization; (Equal), Writing – original draft; (Equal), Writing – review & editing; (Equal). **Johannes Ring**: Conceptualization; (Equal). Writing – original draft. (Equal), Writing – review & editing; (Equal). **Motohiro Ebisawa**: Conceptualization; (Equal), Writing – original draft; (Equal), Writing – review & editing; (Equal). **Guillaume Pouessel**: Conceptualization; (Equal), Writing – original draft; (Equal), Writing – review & editing; (Equal). **Pete Smith**: Conceptualization; (Equal), Writing – original draft; (Equal), Writing – review & editing; (Equal).

## CONFLICT OF INTEREST STATEMENT

MWo has received financial grants from ALK‐Abelló Arneimittel, Mylan Germany GmbH. AF received consultation fees for his trust from Viatris. No personal fee was involved. MWi has been a paid instructor and has received speaker fees for MEDA and Mylan. JR serves as a consultant for Viatris and has received speaker fees from Galderma, Viatris, Bencard, Sanofi and AbbVie. ME has received speaker fees from Mylan and DBV Technologies. GP has received consultation fees from Bausch and Lomb, Viatris, BioProject, and speaker fees from Bausch and Lomb, AI therapeutics, BioProject and Viatris. PS received financial grants from Mylan, GlaxoSmithKline, and Sanofi for being a part of investigator‐initiated studies and has received consultation fees from Sequirus, AstraZeneca, Novartis and Viatris (for being a part of advisory board).

## Supporting information

Supporting Information S1

## Data Availability

Data sharing is not applicable to this review article as the datasets are publicly available.
