# Supplementary material for: Adrenaline auto injectors pharmacokinetic/pharmacodynamic studies and potential consequences for clinical practice
Source: Clin Transl Allergy. 2023 Dec 18;13(12):e12323. doi: 10.1002/clt2.12323 (PMC10728507; doi:10.1002/clt2.12323)
Supplement: Supplementary file 1 — Supporting Information S1 [file CLT2-13-e12323-s001.docx]

**Supplementary Tables**

**Supplementary Table 1**: Included Studies Overview

| **Parameters** |  | **Product A**^26^ | **IM**  **syringe**^26^ | |  | **Product EP**^27^ | **Product EM**^27^ | | **Product J**^27^ | |  | **Product EP**^28^ | | | **IM syringe**^28^ | |  |
| --- | --- | --- | --- | --- | --- | --- | --- | --- | --- | --- | --- | --- | --- | --- | --- | --- | --- |
| **Device delivery system** |  | syringe | syringe | |  | cartridge | syringe | | cartridge | |  | cartridge | | | syringe | |  |
| **Dose (µg)** |  | 300 | 300/500 | |  | 300 | 300 | 500 | | 300 | | |  | 300 | | 300 | |
| **Route** |  | IM^a^/SC^b^ | IM | |  | IM | IM | IM | | IM | | |  | IM | | IM | |
| **Study design** |  | Crossover | | |  | Four way cross-over | | | | |  | Crossover | | | | |  |
| **Sample Size (N)** |  | 30 | | |  | 35 | | | | |  | 35 | | | | |  |
| Cohort 1 |  | 18 | | |  | 15 | | | | |  | 12 | | | | |  |
| Cohort 2 |  | 12 | | |  | 14 | | | | |  | 12 | | | | |  |
| Cohort 3 |  | 0 | | |  | 6 | | | | |  | 11 | | | | |  |
| **Gender** |  |  | | |  |  | | | | |  |  | | | | |  |
| Cohort 1 |  | M (n=18) | | |  | M (n=8)  W (n=8) | | | | |  | M (n=6)  W (n=6) | | | | |  |
| Cohort 2 |  | W (n=12) | | |  | M (n=8)  W (n=7 | | | | |  | M (n=6)  W (n=6) | | | | |  |
| Cohort 3 |  | NA | | |  | M (n=0)  W (n=7) | | | | |  | M (n=5)  W (n=6) | | | | |  |
| **STMD** |  |  | | |  |  | | | | |  |  | | | | |  |
| Cohort 1 |  | 0.59 ±0.18 cm/ 0.59 ± 0.19 cm  (Low STMD) | 0.60 ± 0.17 cm/ 0.44 ±0.10 cm  (Low STMD) | |  | ≥10, <15 mm  (Low STMD) | | | | |  | <15 mm  (Low STMD) | | | | |  |
| Cohort 2 |  | 1.58 ± 0.36 cm  (Moderate STMD) | | |  | ≥15, ≤20 mm  (Moderate STMD) | | | | |  | 15–20 mm  (Moderate STMD) | | | | |  |
| Cohort 3 |  | NA | | NA |  | >20 mm  (High STMD) | | | | |  | >20 mm  (High STMD) | | | | |  |
| **Needle length** | | | | | | | | | | | | | | | | |  |
| Low STMD |  | 10.5 mm | 25.4 mm | |  | 16 mm | 23 mm | 23 mm | | 15 mm | | |  | 16 mm | | 19.4 mm | |
| Moderate STMD |  | 10.5 mm | NA | |  | 16 mm | 23 mm | 23 mm | | 15 mm | | |  | 16 mm | | 27.9 mm | |
| High STMD |  | NA | NA | |  | 16 mm | 23 mm | 23 mm | | 15 mm | | |  | 16 mm | | 39.1 mm | |
| **Needle gauge** | | | | | | | | | | | | | | | | |  |
|  |  | 27 G | 25 G | |  | NA | NA | NA | | NA | | |  | 22 G | | 23 G^c^  Range:  (22 G-27 G) | |
| **BMI** | | | | | | | | | | | | | | | | |  |
|  |  | 18-34 kg/m^2^ | | |  | 20.6-38.1 kg/m^2^ | | | | |  | 18-40 kg/m^2^ | | | | |  |
| ^a^Adrenaline injected via Product A reached the intramuscular layer in low STMD participants; ^b^Adrenaline injected via Product A reached the subcutaneous layer in moderate STMD participants  ^c^Needle gauge expressed as median  **Abbreviations.** G, gauge; IM, intramuscular; M, men; N, sample size; NA, not available; STMD, skin-to-muscle distance; W, women  **Note: For Product J vs. IM syringe**, device delivery system and dose, cartridge (300 µg) vs. syringe (300 µg). Adrenaline dose from both devices have been injected IM in a crossover manner | | | | | | | | | | | | | | | | |  |

**Supplementary Table 2.** Summary of the PK parameters after adrenaline administration via Product A and IM syringe^26^

| **PK Parameters** |  | **Low STMD** | | | | **Moderate STMD** |
| --- | --- | --- | --- | --- | --- | --- |
| Dose (µg) |  | Product A 300 µg | Product A 300 µg | IM syringe 300 µg | IM syringe 500 µg | Product A 300 µg |
| Gender/Location of injection |  | M (mid-AL thigh) | M (distal-AL thigh) | M (mid-AL thigh) | M (mid-AL thigh) | W (distal-AL thigh) |
| C_peak0-20 min_ (pg/mL) |  | 377.0 | 353.9 | 222.6 | 401.2 | 440.0 |
| T_peak0-20 min_  (min) |  | 0.21±0.12 | 0.15±0.08 | 0.18± 0.13 | 0.15± 0.11 | 0.24±0.10 |
| AUC_0-20 min_  (min*pg/mL) |  | 4158^a^ | 4104^a^ | 2310^a^ | 4554^a^ | 3354^a^ |
| AUC_0-240 min_  (min*pg/mL) |  | 27552^a^ | 28386^a^ | 30198^a^ | 46632^a^ | 40662^a^ |
| **Notes:**  Low STMD expressed as mean and standard deviation values as follows: 5.9±1.8 mm; 6.0±1.7 mm; 5.9±1.9 mm; 4.4±1.0 mm  Moderate STMD expressed as mean and standard deviation values as follows: 15.8±3.6 mm  All the given values are expressed as mean.  ^a^The values for AUC_0-20 min*pg/mL_ and AUC_0-240 min*pg/mL_ have been obtained by multiplying values with 60 min to convert hours to minutes  **Abbreviations.** AUC_0-20 min_, area under the concentration-time curve from time zero until 20 min; AUC_0-240 min_, area under the concentration-time curve from time zero until 240 min; C_peak_, peak plasma drug concentration within the first 20 min; M, men; STMD, skin-to-muscle distance; T_peak0-20 min_, time for peak adrenaline plasma concentration observed within the first 20 min; W, women | | | | | | |

**Supplementary Table 3.** Summary of the PK parameters of adrenaline administered via Product EM 300 μg and 500 μg, Product EP, and Product J^27^

| **Low STMD^a^ (Cohort 1)** | | | | | | | | | | | | |
| --- | --- | --- | --- | --- | --- | --- | --- | --- | --- | --- | --- | --- |
| PK Parameters |  | Product EP | |  | Product EM 300 μg | |  | Product EM 500 μg | |  | Product J | |
|  |  | M | W |  | M | W |  | M | W |  | M | W |
| C_max_ (pg/mL) |  | 444.4 | 383.2 |  | 281.7 | 318.9 |  | 386.0 | 324.6 |  | 287.7 | 332.0 |
| T_max_ (min) |  | 22.5 | 20.0 |  | 55.0 | 40.0 |  | 50.0 | 50.0 |  | 40.0 | 30.0 |
| AUC_0- 10 min_  (pg*min/mL) |  | 1268.1 | 1087.3 |  | 678.7 | 647.6 |  | 593.2 | 1175.3 |  | 702.5 | 1120.2 |
| AUC_0-last_  (pg*min/mL) |  | 22726 | 21267 |  | 21738 | 20690 |  | 31118 | 28513 |  | 22479 | 21884 |
| **Moderate STMD^b^ (Cohort 2)** | | | | | | | | | | | | |
| PK Parameters r |  | Product EP | |  | Product EM 300 μg | |  | Product EM 500 μg | |  | Product J | |
|  |  | M | W |  | M | W |  | M | W |  | M | W |
| C_max_ (pg/mL) |  | 333.2 | 414.7 |  | 226.6 | 213.9 |  | 297.3 | 400.1 |  | 213.3 | 243.2 |
| T_max_ (min) |  | 16.0 | 20.0 |  | 60.0 | 75.0 |  | 55.0 | 50.0 |  | 40.0 | 90.0 |
| AUC_0-10 min_ (pg*min/mL) |  | 1075.1 | 1279.2 |  | 811.3 | 268.5 |  | 1085.5 | 454.9 |  | 629.6 | 287.5 |
| AUC_0-last_  (pg*min/mL) |  | 22307 | 28507 |  | 18465 | 21225 |  | 26458 | 32720 |  | 18073 | 26002 |
| **High STMD^c^ (Cohort 3)** | | | | | | | | | | | | |
| PK Parameters |  | Product EP | |  | Product EM 300 μg | |  | Product EM 500 μg | |  | Product J | |
|  |  | M | W |  | M | W |  | M | W |  | M | W |
| C_max_ (pg/mL) |  | NA | 363.3 |  | NA | 228.2 |  | NA | 543.4 |  | NA | 276.3 |
| T_max_ (min) |  | NA | 40.0 |  | NA | 45.0 |  | NA | 60.0 |  | NA | 50.0 |
| AUC_0- 10 min_  (pg*min/mL) |  | NA | 842.8 |  | NA | 415.8 |  | NA | 810.8 |  | NA | 516.3 |
| AUC_0-last_  (pg*min/mL) |  | NA | 30383 |  | NA | 20507 |  | NA | 51065 |  | NA | 23896 |
| ^a^Cohort 1, STMD measuring ≥10, <15 mm  ^b^Cohort 2, STMD measuring ≥15, ≤20 mm  ^c^Cohort 3, STMD measuring >20 mm  **Abbreviations.** AUC_0-10 min_, area under the concentration-time curve from time zero until 10 min; AUC_0-last,_ area under the concentration-time curve from time zero to the last measured concentration, C_max_, peak plasma drug concentration; M, men; NA, not available; STMD, skin-to-muscle distance; T_max_, time to C_max_; W, women | | | | | | | | | | | | |
|  | | | | | | | | | | | | |

**Supplementary Table 4.** Summary of the PK parameters of adrenaline administered via Product EP and IM syringe^28^

|  |  | **Low STMD^a^** | | | |  | **Moderate STMD^b^** | | | |  | **High STMD^c^** | | | |
| --- | --- | --- | --- | --- | --- | --- | --- | --- | --- | --- | --- | --- | --- | --- | --- |
| PK Parameters (mid-AL thigh) |  | Product EP | | IM syringe | |  | Product EP | | IM syringe | |  | Product EP | | IM syringe | |
|  |  | M | W | M | W |  | M | W | M | W |  | M | W | M | W |
| C_max_ (pg/mL) |  | 400.0 | 640.0 | 250.0 | 360.0 |  | 480.0 | 520.0 | 320.0 | 370.0 |  | 420.0 | 630.0 | 270.0 | 510.0 |
| T_max_ (min)^d^ |  | 17.0 | 5.0 | 50.0 | 40.0 |  | 4.5 | 15.0 | 49.5 | 45.0 |  | 30.0 | 24.5 | 50.0 | 50.0 |
| AUC_p(0-30 min)_^e^ |  | 2.09 | | | |  | 1.64 | | | |  | 2.90 | | | |
| AUC_0-last_  pg*min/mL |  | 27300 | 28800 | 20100 | 28800 |  | 20900 | 31700 | 25700 | 28100 |  | 31100 | 40300 | 22600 | 30600 |
| PK Parameters (distal-AL thigh) |  | Product EP | | IM syringe | |  | Product EP | | IM syringe | |  | Product EP | | IM syringe | |
|  |  | M | W | M | W |  | M | W | M | W |  | M | W | M | W |
| C_max_ (pg/mL) |  | NA | NA | NA | NA |  | 360 | 490 | NA | NA |  | 300 | 460 | NA | NA |
| T_max_ (min)^d^ |  | NA | NA | NA | NA |  | 29.5 | 25 | NA | NA |  | 30 | 9 | NA | NA |
| AUC_0-last_  pg*min/mL |  | NA | NA | NA | NA |  | 22500 | 26900 | NA | NA |  | 25100 | 37400 | NA | NA |
| ^a^STMD measuring <15 mm  ^b^STMD measuring, 15–20 mm  ^c^STMD measuring, >20 mm  ^d^The given values are expressed as median  ^e^The given values are expressed as geometric mean ratio of Product EP vs. IM syringe  **Note:** The given values for AUC_0-t_ have been converted from ng*min/mL and converted to pg*min/mL for uniformity  **Abbreviations.** AUC_p (0-30 min)_, partial area under the concentration-time curve over first 30 min; AUC_0-last,_ area under the concentration-time curve from time zero to the last measurable concentration; C_max_, peak plasma drug concentration; M, men; PK, pharmacokinetics; NA, not available; STMD, skin-to-muscle distance; T_max_, time to C_max_; W, women | | | | | | | | | | | | | | | |

**Supplementary Table 5.** Summary of the PK parameters after adrenaline administration via Product J and IM syringe^29^

| **PK Parameters** |  | **Low STMD^a^** | |  | **Moderate STMD^b^** | |  | **High STMD^c^** | |
| --- | --- | --- | --- | --- | --- | --- | --- | --- | --- |
|  |  | **Product J** | **IM syringe** |  | **Product J** | **IM syringe** |  | **Product J** | **IM syringe** |
| C_max_ (pg/mL) ^d^ |  | 1.07 (0.84–1.37) | |  | 0.78 (0.54–1.13) | |  | 1.13 (0.81–1.58) | |
| T_max_ (min)^e^ |  | 30 | NA |  | 12 | NA |  | 60 | NA |
| AUC_0-30 min_^d^ |  | 1.16 (0.86–1.58) | |  | 0.85 (0.59–1.25) | |  | 0.66 (0.39–1.12) | |
| AUC_0-last_^d^ |  | 1.23 (1.04–1.46) | |  | 0.99 (0.82–1.19) | |  | 1.92 (1.58–2.33) | |
| ^a^STMD measuring <15 mm  ^b^STMD measuring ≥15 mm–≤20 mm  ^c^STMD measuring >20 mm  ^d^The given data is expressed in terms of geometric least square mean of ratio Product J/IM syringe with 90% confidence intervals  ^e^The given data is expressed as median  **Abbreviations**: AUC_0-30 min,_ area under the concentration-time curve from time zero until 30 min; AUC_0-last,_ area under the concentration-time curve from time zero until to the last measured concentration; C_max_, peak plasma drug concentration; NA, Not Available; T_max_, time to C_max;_ | | | | | | | | | |
